# Epithelial-mesenchymal transition induction is associated with augmented glucose uptake and lactate production in pancreatic ductal adenocarcinoma

**DOI:** 10.1186/s40170-016-0160-x

**Published:** 2016-10-17

**Authors:** Menghan Liu, Lake-Ee Quek, Ghazal Sultani, Nigel Turner

**Affiliations:** 1 Department of Pharmacology, School of Medical Sciences, University of New South Wales, Sydney, NSW Australia; 2 Charles Perkins Centre, School of Mathematics and Statistics, The University of Sydney, Sydney, NSW 2006 Australia

**Keywords:** Epithelial-mesenchymal transition, Tumor metabolism, Pancreatic adenocarcinoma, Glucose metabolism, ^13^C Metabolomics

## Abstract

**Background:**

Pancreatic ductal adenocarcinoma (PDAC) is a common malignancy with dismal prognosis. Metastatic spread and therapeutic resistance, the main causes of PDAC-related mortalities, are both partially underlined by the epithelial-mesenchymal transition (EMT) of PDAC cells. While the role of Warburg metabolism has been recognized in supporting rapid cellular growth and proliferation in many cancer types, less is known about the metabolic changes occurring during EMT, particularly in the context of PDAC.

**Results:**

In the current study, experimental models of EMT were established in the Panc-1 cell line of human PDAC via exposure to two physiologically relevant EMT inducers (tumor necrosis factor-α and transforming growth factor-β) and the metabolic consequences examined. The two EMT models displayed similar alterations in the general metabolic profile including augmented glucose uptake and lactate secretion as well as the lack of change in oxidative metabolism. Examination of molecular markers revealed differences in the pathways underlying the metabolic rewiring. ^13^C-Glucose tracer data confirmed that a major portion of accumulated lactate was derived from glucose, but subsequent flux analysis suggested involvement of non-canonical pathways towards lactate production.

**Conclusions:**

Our results characterize the metabolic reprogramming occurring during PDAC cell EMT and highlight the common changes of increased glucose uptake and lactate secretion under different EMT conditions. Such insight is urgently required for designing metabolic strategies to selectively target cells undergoing EMT in PDAC.

**Electronic supplementary material:**

The online version of this article (doi:10.1186/s40170-016-0160-x) contains supplementary material, which is available to authorized users.

## Background

With a 5-year survival rate of less than 5 %, pancreatic ductal adenocarcinoma (PDAC) is the most lethal common malignancy facing human society [1]. Surgical resection provides the only chance of cure, but most patients are diagnosed at an advanced stage where local invasion or distant metastasis has occurred [2, 3]. The inability to undergo surgical resection and therapeutic resistance to currently available chemotherapies together contributes to pancreatic cancer’s poor prognosis [2, 4].

The propensity for both metastasis and therapeutic resistance in tumors has been proposed to be associated with the epithelial-mesenchymal transition (EMT) [5]. EMT is a trans-differentiation process active in embryogenesis and tissue repair where epithelial cells acquire the phenotype and gene expression patterns of mesenchymal cells [5]. In the context of tumor development, pathological EMT occurs when neoplastic cells of epithelial origin transiently switch to a mesenchymal-like phenotype in order to gain increased mobility, invasiveness, stemness, and apoptotic resistance [6, 7]. Overexpression of EMT-inducing transcription factors Snail and Slug have been reported in human PDAC samples and cell lines [8]. Disseminated cells from early-stage PDAC tumors were also found to express higher levels of mesenchymal markers and EMT-inducing transcription factors, paired with enhanced survival and self-renewal properties, compared to the bulk of cells in the primary tumor site [9]. Furthermore, pancreatic cancer cells that survive after gemcitabine treatment, the frontline chemotherapeutic intervention, tend to acquire EMT-like phenotypes [10–12].

It is well-recognized that the development of tumors requires not only the ability to proliferate uncontrollably, but also altered metabolic programs to sustain the rapid expansion [13–15]. While there are changes common to multiple cancer types such as upregulated glucose uptake and lactate production, known as the Warburg effect, the metabolic profiles of individual tumors and tumors at different stages of development also possess unique features due to the heterogeneous nature of cancers. Studies in recent years have reported that PDAC tumors take up increased amounts of glucose to fuel biosynthetic processes [16], display elevated glutaminolysis to maintain redox balance [17] and scavenge fatty acids as well as amino acids from extracellular space to synthesize macromolecules such as lipids and proteins [18, 19]. These metabolic adaptations are the results of oncogenic signaling active in PDAC and the tumor microenvironment modulation, which collectively meet the cell’s demand to accumulate biomass and proliferate.

EMT represents a series of major phenotypic alterations and signals for a change in cellular demand from rapid proliferation to survival and metastasis. It is therefore plausible that cellular metabolism is reshaped to reflect and support the new cell state. Indeed, research exploring the possible metabolic reprogramming events associated with EMT has emerged over the past 5 years describing alterations of multiple metabolic enzymes involved in a range of metabolic pathways upon EMT induction in various cancer types [20–27]. Metabolic reprogramming could also feedback into the EMT machinery as manipulations of affected metabolic enzymes have partially blocked or potentiated the induction of EMT [22, 23, 27]. Intriguingly, EMT-related metabolic changes reported in different cancer types appear to be disparate, highlighting the plasticity and context dependency of cancer metabolism. Considering the importance of EMT in underlying PDAC-related mortalities, we investigated the metabolic consequences of inducing EMT in a commonly used PDAC cell line, Panc-1, with tumor necrosis factor-α (TNFα) and transforming growth factor-β (TGFβ). Here, we report that EMT states induced by both factors are accompanied by increased glucose uptake and lactate secretion and propose possible mechanisms for the observed metabolic reprogramming.

## Methods

### Antibodies and reagents

Recombinant human TNFα and TGFβ were purchased from R&D Systems (Minneapolis, MN, USA). Antibodies used are listed as follows: IkB, phospho-Smad2/3, total-Smad2/3, Snail, vimentin, lactate dehydrogenase-A, fatty acid synthase, stearoyl-CoA desaturase-1, phospho-acetyl-CoA carboxylase, total-acetyl-CoA carboxylase (Cell signaling, MA, USA); beta-actin, E-cadherin, N-cadherin (Santa Cruz, TX, USA); hexokinase II, pyruvate kinase isoform 2, fructose-bisphophotase-2, total OXPHOS Human WB Antibody Cocktail (Abcam, Cambridge, UK); Zeb1 (ProSci, CA, USA); and fructose-bisphophotase-1 (Abgent, Wuxi, China). All other reagents were from Sigma-Aldrich (MO, USA) unless stated otherwise.

### Cell culture

Panc-1 cells from ATCC were cultured in Dulbecco’s modified Eagle’s medium containing 4.5 g/L glucose supplemented with 10 % fetal bovine serum and penicillin-streptomycin. Panc-1 cells were treated with TNFα and TGFβ at 40 and 10 ng/ml, respectively, for 72 h unless otherwise specified.

### Western blotting

Cells (1 × 10^6^) growing on six-well plates were lysed in RIPA buffer containing protease inhibitors as described previously [28]. After denaturation, samples were resolved by 10 % polyacrylamide gel electrophoresis (187 V, 1 h) and transferred to PVDF membranes (65 V, 65 min). For immunoblotting, membranes were blocked in 5 % skim milk, incubated with primary antibodies overnight at 4 °C, washed with Tris-buffered saline containing Tween 20 and incubated with secondary antibodies for an hour. After washing, the membranes were developed with enhanced chemiluminescence reagents (Western Lighting Plus-ECL, Perkin Elmer, MA, USA) and visualized under Las4000 imager (GE Healthcare, WI, USA). Densitometry was performed using ImageJ by obtaining the optical density of each band.

### Quantitative PCR

Total RNA was extracted using the Roche High Pure RNA Extraction kit according to manufacturer’s instructions (Roche, Basel, Switzerland). RNA (1 μg) was then reverse-transcribed using the Roche Transcriptor first-strand cDNA synthesis kit. The resulting cDNA was mixed with primers (primer sequences sourced from Sigma KiCqstart or the Primer Bank [29], details in Additional file 1: Table S1) and SYBR-Green (Roche, Basel, Switzerland) in 384- or 96-well plates. Quantitative PCR was performed using Roche 480 Light Cycler to obtain Ct values for each gene of interest and a housekeeper beta-actin. Analysis was conducted using the ΔΔCT method.

### Wound healing assay

Panc-1 cells were seeded in a six-well plate and grown to 50 % confluence over 2 days in the presence of TNFα, TGFβ, or both. Scratch wounds were created by scraping confluent cell monolayers with a sterile pipette tip on three sites on each well. The cells were then incubated under normal conditions with refreshed media containing TNFα, TGFβ, or both under the Nikon Tie inverted time-lapse microscope for 48 h. Migration at 24 and 48 h was quantified by measuring the distance between two moving borders of the cells from each scratch. Values from the three wounds on each of the triplicate wells were averaged, and three independent experiments were carried out (Additional file 2: Figure S1).

### Glucose uptake assay

Glucose uptake was assessed using the glucose analogue 2-deoxyglucose (2-DG) which is taken up into the cell in the same manner as glucose but once phosphorylated cannot be further metabolized and is therefore trapped in the cell. Cytochalasin B (25 μM) was applied to a few wells for 15 min before the assay and during the assay to give a measure of background glucose uptake.

After washing with PBS, cells in six-well plates were incubated in Ringer’s solution (140 mM NaCl, 20 mM HEPES, 5 mM KCl, 2.5 mM MgSO4, 1.2 mM CaCl_2_, pH 7.4) containing 10 μM 2-DG and 0.5 μCi/ml radio-labelled ^3^H-2-DG for exactly 8 min. Following incubation, cells were washed with cold PBS and lysed in 1 M NaOH. The amount of ^3^H radioactivity in lysates was counted using a beta-counter (Tri-Carb liquid scintillation counter, Perkin Elmer, MA, USA) from which background was subtracted. Protein concentrations of lysates were measured using BCA assay (Pierce BCA protein assay kit, Thermo Fisher Scientific, MA, USA) for normalization.

### Lactate assay

Lactate concentrations in cell culture media were determined in a reaction mixture containing hydrazine hydrate (0.4 M, pH 9.0), EDTA (10 mM, pH 9.0), and NAD^+^ (0.5 mM). Samples and standards were added into 96-well plates followed by lactate dehydrogenase (10 units/well), and the amount of lactate was assessed by measuring the amount of NADH formed at 340 nm after 2 h of incubation at 37 °C (a time point when lactate conversion is complete).

### Basal oxygen consumption

Cellular oxygen consumption was measured by the XF24 Analyzer (Seahorse Bioscience, MA, USA) following manufacturer’s protocols. Cells were plated in five replicates in 24-well Seahorse assay plates and subjected to treatments in growth media for 72 h. Prior to the assay, media was changed to Seahorse assay media with the addition of 4.5 g/L glucose and 2 mM glutamine, pH 7.4. The basal oxygen consumption was measured over 3 min for three times with 1 min mixing in between the measurements. Data were analyzed using the Seahorse software. Basal oxygen consumption was normalized to protein concentration determined by BCA method.

### Glucose oxidation

Glucose oxidation was measured in cells seeded in six-well plates (1 × 10^6^ cells per well). Briefly, cells were washed with PBS and incubated in DMEM containing 1 g/L d-glucose and 2 μCi/ml ^14^C-glucose for 1 h at 37 °C. After incubation, the culture media was added to 1 M perchloric acid and the CO_2_ released was absorbed in 1 M NaOH solution over 2 h. The CO_2_ produced was quantified by counting ^14^C content in the NaOH solution using a beta-counter (Tri-Carb liquid scintillation counter, Perkin Elmer, MA, USA).

### Lipid production from glucose

Cells from glucose oxidation experiments were collected and snap-frozen to measure the amount of ^14^C incorporated into lipids over the duration of the assays. For total lipid extraction, chloroform:methanol (volume ratio 2:1) was added to the cells followed by 3 h of rotation at room temperature. Sulfuric acid (2 M) was then added to achieve phase separation, and the organic solvent layer containing lipid was aspirated into a glass vial and dried under nitrogen gas. The lipid was dissolved in ethanol and its ^14^C content counted using a beta-counter and normalized for protein concentration of the cell lysate.

### ^13^C-Glucose tracer experiments

Panc-1 cells were seeded in a six-well plate and grown to 1 × 10^6^ cells over 72 h in the presence of TNFα, TGFβ, or both. To start the tracer experiment, culture media were refreshed to glucose-free DMEM containing 4.5 g/L [U-^13^C]-glucose (Sigma-Aldrich, Castle Hill, Australia) and the respective cytokines. Fifty microliters of culture medium was sampled at the start and at every hour for 5 h. Samples were placed on ice and then stored in a −30 °C freezer until derivatization. Cell density by protein content was determined using three concurrent cell culture replicates containing normal glucose.

Polar intracellular metabolites were harvested from cells of the same tracer experiment at the fifth hour. Briefly, cells were washed and quenched using ice-cold isotonic NaCl, then the metabolite were extracted using 5 ml of 50 vol.% methanol/water at −40 °C [30]. Polar metabolites were retained by vortexing the extraction mix with equal volume of chloroform and subsequently discarding the organic phase. The aqueous phase was dried by SpeedVac and then stored in a −30 °C freezer until derivatization.

Calibration standards for glucose, lactate, and pyruvate were prepared. With a starting concentration of 20 mM glucose, 10 mM lactate, and 2 mM pyruvate, four sets of standard concentrations were prepared by twofold serial dilution. Standards were stored in freezer until derivatization.

### Metabolite sample preparation and GC-MS

Ten microliters of the thawed culture medium or calibration standards was combined with 10 μl of succinic acid-d6 (10 mM) in a glass vial and was evaporated to dryness by SpeedVac. Dried samples were resuspended in 15 μl of pyridine containing 20 mg/ml methoxyamine HCl, and then incubated at 80 °C for 1 h. Fifteen microliters of acetic anhydride was added, followed by another hour of incubation at 80 °C. Once cooled to room temperature, 50 μl of 1-butanol and 10 μl of ethyl chloroformate were added in succession, with each step followed by brief vortexing. Samples were kept at room temperature for 5 min before being transferred into 600 μl microcentrifuge tubes. Eighty microliters of chloroform was added, followed by 10–15 mg of sodium hydrogen carbonate solids and 75 μl of saturated sodium hydrogen carbonate solution. The organic and aqueous phases were mixed by pipetting. After the bubbling had ceased, a further 150 μl of saturated sodium hydrogen carbonate solution was added. After brief vortexing, samples were centrifuged at 500*g* for 5 min. Approximately 70 μl of the chloroform (bottom) phase was transferred into GC-MS vials.

Dried intracellular metabolites were derivatized by silylation [31]. Fofty microliters of pyridine containing 20 mg/ml methoxyamine HCl was added, followed by an hour of incubation at 80 °C. Thirty microliters of MTBSTFA + 1 % t-BDMCS was then added, followed by another hour of incubation at 80 °C. Derivatized samples were then transferred into GC-MS vials.

Derivatized metabolites were analyzed by GC-MS using a HP-5ms capillary column (0.25 mm i.d. × 30 m × 0.25 μm; Agilent J&W, Agilent Technologies, CA, USA) installed in an Agilent HP 6890-5973 gas chromatography/mass selective detector. The injection volume was 1 μL in splitless mode with an inlet temperature of 250 °C. Helium flow was controlled at 1.1 ml/min. The MS was operated in electron ionization mode at 70 eV. The temperatures of the source, quadrupole, and the transfer line were set at 150, 230, and 250 °C, respectively. For extracellular metabolites, the GC temperature program was set at 100 °C for 2 min, ramped at 15 °C/min to 150 °C and at 40 °C/min to 325, and finally held for 1.3 min. Ions for pyruvate (*m*/*z* 142–145), lactate (*m*/*z* 115–118), and glucose (*m*/*z* 314–318) were quantified by selective ion monitoring mode. For intracellular (silylated) metabolites, the GC temperature program was set at 70 °C for 2 min, ramped at 4 °C/min to 200 °C and at 15 °C/min to 290 °C, and finally held for 6 min. Ions for pyruvate, lactate, and TCA cycle metabolites were quantified by selective ion monitoring mode. GC-MS peak integration and correction for natural isotope mass interference were performed using MATLAB scripts.

### ^13^C Flux analysis

A flux analysis approach was used to determine the proportion and rate of lactate produced from glucose and from non-glucose sources. A simplified glycolysis pathway was used to map glucose carbons to pyruvate and lactate (Fig. 4a). Non-glucose-derived carbon sources were assumed to mix with the glucose carbons at the pyruvate node. OpenFLUX was used to generate the metabolite and atom balance models required for flux analysis (Additional file 3: Figure S2) [32]. The non-stationary enrichment of extracellular lactate and pyruvate was modelled by a forward Euler method that described the accumulation dynamics of labelled lactate and pyruvate over time (Additional file 4). Sensitivity analysis of estimated flux parameters was performed using a Monte Carlo approach. Due to the lack of enrichment data of glycolytic intermediates, intracellular isotopic steady-state was assumed.

Flux estimation was performed by constraint-based least squares fitting of experimental data using MATLAB R2012a (MathWorks, Natick, MA). This research includes computations using the Linux computational cluster Katana supported by the Faculty of Science, UNSW, Australia.

### Statistical analysis

All results are expressed as means ± standard error of the mean (SEM). Results were analyzed by one-way ANOVA with a Bonferroni post hoc test applied to compare every treatment group to the control. Differences with *p* < 0.05 were deemed statistically significant.

## Results

### EMT was experimentally induced upon TNFα and TGFβ treatments

To study the metabolic alterations associated with EMT in PDAC, we first sought to establish models of experimentally induced EMT in a human PDAC cell line. In vivo, EMT is induced by a range of factors in the tumor microenvironment among which TNFα and TGFβ have been shown to induce EMT in multiple cancer types [33–37]. PDAC is known to have a dense stromal component containing myofibroblasts as well as infiltrating immune cells, and a hyperactive TGFβ signaling in a subset of these tumor cells [38, 39]. We therefore modelled EMT by treating Panc-1 cells, a commonly used human PDAC cell line harboring the prevalent KRAS mutation, with the principal cytokine secreted by macrophages (TNFα), or the stromal-derived growth factor TGFβ or a combination of both factors.

**Fig. 1:**
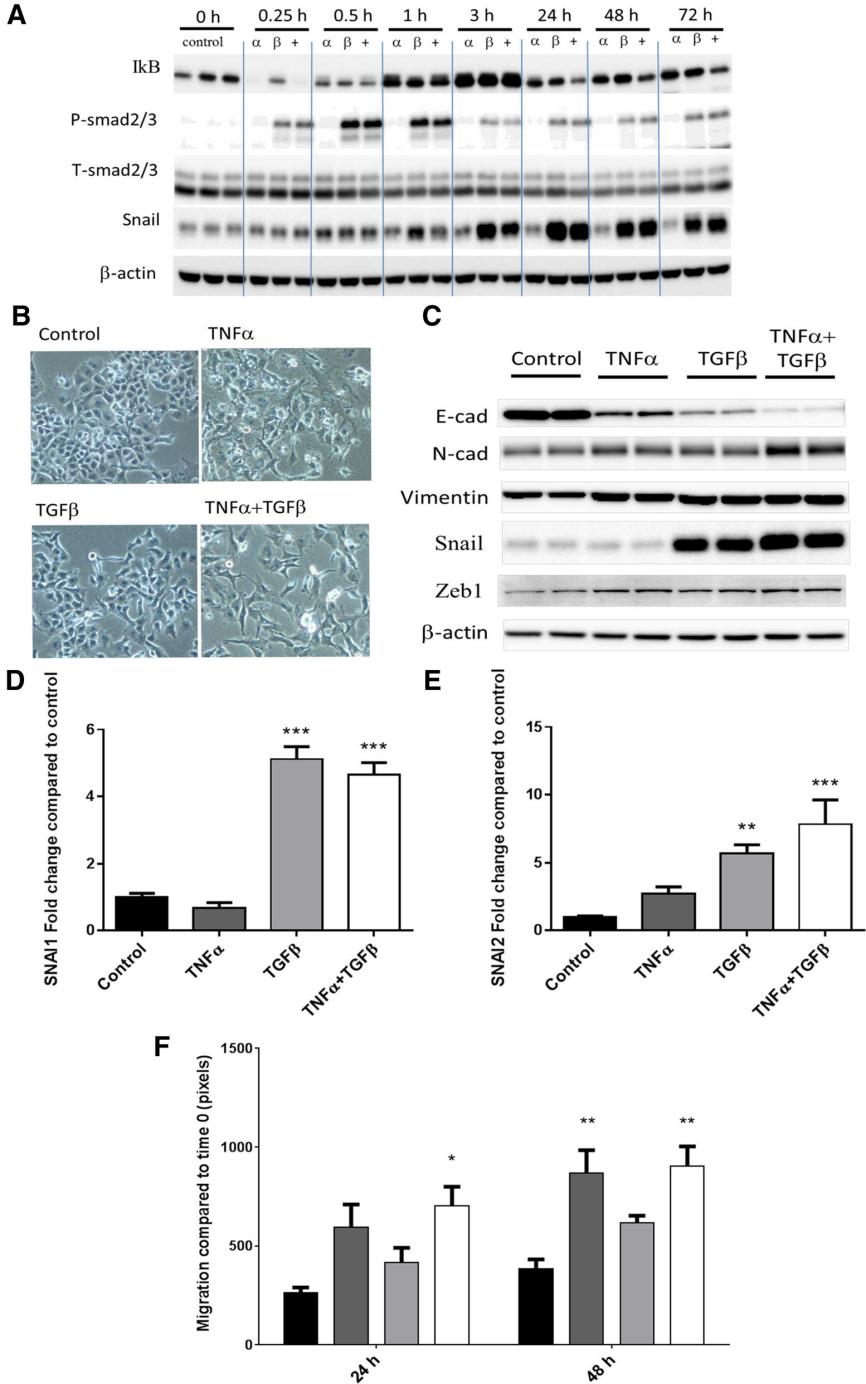
EMT is induced upon TNFα and TGFβ treatments. Panc-1 cells were cultured in the presence of 40 ng/ml TNFα (α) or 10 ng/ml TGFβ (β) or both (+) for 72 h. **a** Western blot detection of IkB, phospho-Smad2/3, total-Smad2/3, and Snail at 0, 0.25, 0.5, 1, 3, 24, 48, and 72 h time points. β-actin was used as a loading control. **b** Cell morphology under bright-field microscopy at the end of incubation. **c** Western blot detection of E-cadherin, N-cadherin, vimentin, and Snail. β-actin was used as a loading control. **d**, **e** Measurements of SNAI1 and SNAI2 mRNA levels by qPCR. β-actin was used as a housekeeper. **f** Cells were seeded onto six-well plates and allowed to grow to 50 % confluence in the presence of added factors for 2 days. The monolayer was wounded with a plastic tip and monitored under bright-field microscope for 48 h with continued treatments with images taken at 0, 24, and 48 h. Cell migration was quantified by assessing percentage wound closure using the ImageJ software. Results are shown as mean ± SEM with *n* = 3. *p<0.05, **p < 0.01, ***p < 0.001

While both TNFα and TGFβ can induce EMT [33, 40], these two cytokines were reported to work through different pathways [41, 42], which we initially sought to confirm in the Panc-1 cell line. TNFα mainly signals through the NFkB pathway, with IkB degradation indicative of the activation of this pathway [41]. The canonical signaling pathway of TGFβ, on the other hand, involves Smad2/3 and 4. The phosphorylation of Smad2 or 3 enables the formation of heterodimers with Smad4, which translocate to the nucleus to regulate gene expression [42]. The degradation of IkB and phosphorylation of Smad2/3 were both seen clearly at the 0.25-h time point (Fig. 1a), indicating the rapid activation of TNFα and TGFβ pathways. The signaling events were sustained for a number of hours before inhibitory signals overrode. Snail is one of the major EMT-inducing transcription factors orchestrating the coordinated changes in gene expression during the transition [5]. Figure 1a showed that the Snail level started to accumulate after 1 h of TGFβ treatment, peaked at 24 h, and remained elevated up to 72 h.

Despite engaging different signaling pathways, at the end of the 72-h incubation, morphological changes resembling EMT was clearly observed in all treatment groups, with cells becoming spindle-shaped and scattered compared to cobble stone-shaped controls (Fig. 1b). Molecular characterization revealed that the protein level of epithelial marker E-cadherin was reduced with all treatments (*p* < 0.001), while those of mesenchymal markers N-cadherin (*p* < 0.01 for the combined treatment) and vimentin (*p* < 0.01 for all treatments) were increased (Fig. 1c). The profound increase of Snail (encoded by SNAI1) was seen at the protein and RNA levels when TGFβ was added (Fig. 1c, d). For other prominent members of the EMT-inducing transcription factor family, all treatments resulted in upregulation of Zeb1 at the protein level (*p* < 0.01) and Slug (encoded by SNAI2) was increased by TGFβ at the transcript level, with a similar trend observed for TNFα (Fig. 1c, e). The induction of EMT was further confirmed by an increased migration of Panc-1 cells following treatment for 48 h (Fig. 1f). Overall, robust EMT in the Panc-1 cell line was induced by the addition of TNFα and/or TGFβ to the culture media for up to 72 h.

### Glycolysis is enhanced with TNFα- and TGFβ-induced EMT

**Fig. 2:**
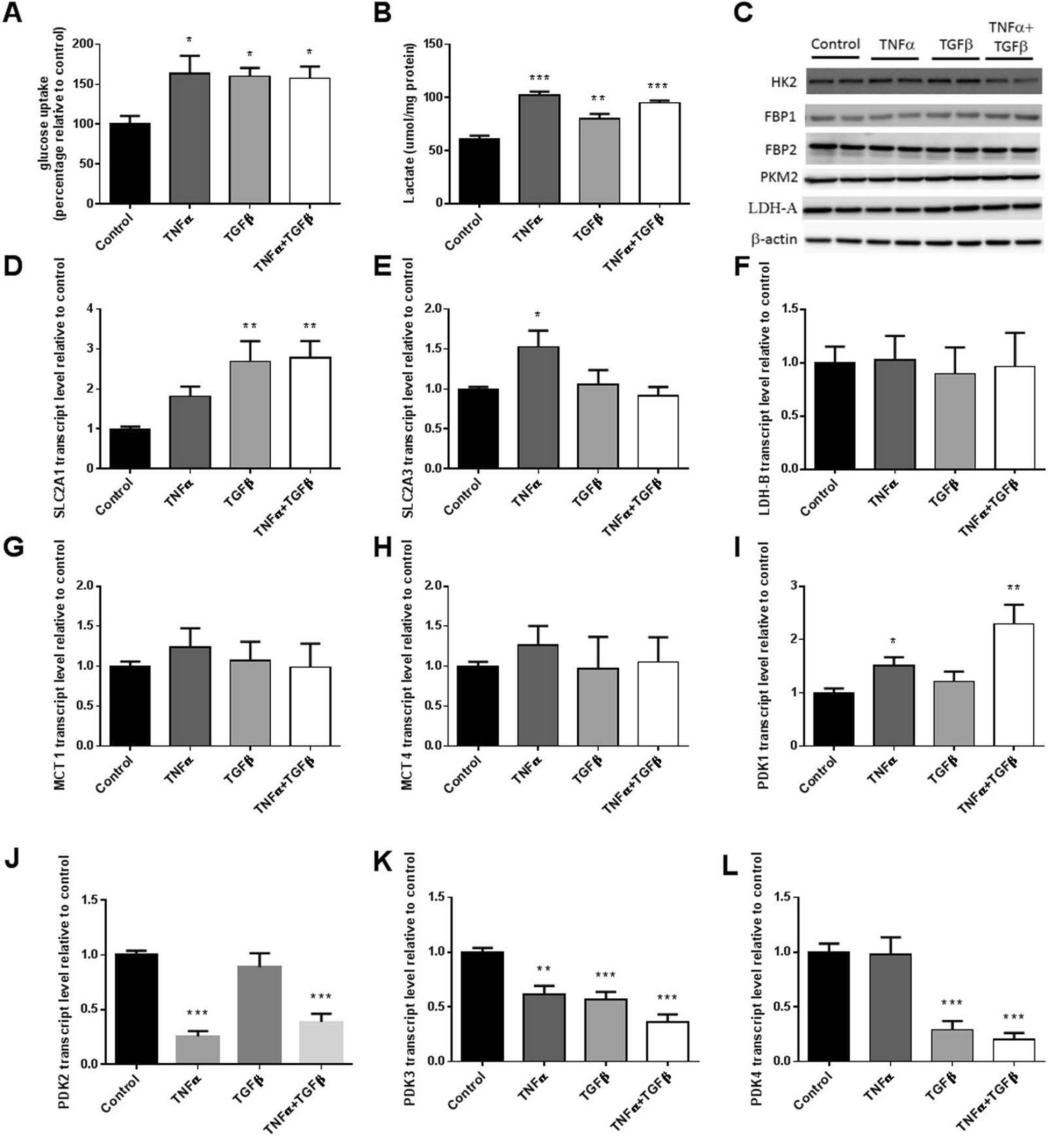
Glycolysis was promoted upon TNFα- and TGFβ-induced EMT. Panc-1 cells were first cultured in the presence of 40 ng/ml TNFα or 10 ng/ml TGFβ or both for 72 h to induce EMT. **a** Rate of glucose uptake was measured using the non-metabolisable glucose analogue ^3^H-2-deoxy-glucose tracer over an 8-min period. The amount of ^3^H radiation trapped in the cells were counted using a beta-counter. **b** Lactate assay was performed on cell culture media collected after 72 h from the respective groups. **c** Western blot detection of hexokinase II (HK2), fructose-1,6-bisphosphotase1 (FBP1), fructose-1,6-bisphosphotase 2 (FBP2), pyruvate kinase isoform 2 (PKM2) and lactate dehydrogenase-A (LDH-A). β-actin was used as a loading control. **d-l** qPCR measurements of SLC2A1 (encoding Glut1), SLC2A3 (encoding Glut3), lactate dehydrogenase B (LDH-B), monocarboxylate transporter 1, (MCT1), monocarboxylate transporter 4 (MCT4) and pyruvate dehydrogenase kinase 1-4 (PDK 1-4) transcripts using β-actin as a housekeeper. Results are shown as mean ± SEM with *n* = 5 (**a**) or *n* = 3 (**b-l**). *p < 0.05, **p < 0.01, ***p < 0.001

Cancer cells are known to exhibit increased glycolytic activity and lactate production compared to their normal counterparts even in the presence of oxygen, a phenomenon described as the Warburg effect. Changes in the glycolytic pathways were therefore investigated upon EMT induction in Panc-1 cells. TNFα, TGFβ, and a combination of the two all led to a nearly twofold increase in glucose uptake (Fig. 2a). The treatments also increased extracellular lactate concentrations, secreted by the cells over 3 days, to varying extents (+33–60 %, *P* < 0.05) (Fig. 2b). Elevated glucose uptake rates were accompanied by increased expression of glucose transporters with Glut1 increased by TGFβ and Glut3 increased by TNFα (Fig. 2d, e). Levels of glycolytic enzymes involved in glucose phosphorylation (hexokinase II (HK2)), pyruvate production (pyruvate kinase isoform 2 (PKM2)), lactate production (lactate dehydrogenase A and B (LDH-A and LDH-B)), and secretion (monocarboxylic acid transporter 1 and 4 (MCT1 and MCT4)) were also examined to elucidate possible mechanisms of increased lactate production. While HK2 increased modestly in response to TNFα (40 %, *p* < 0.05), LDH-A decreased slightly with TNFα treatment (22 %, *p* < 0.05) and PKM2 increased with the combined treatment (14 %, *p* < 0.01), the levels of other glycolytic enzymes remained the same (Fig. 2c, f–h). The expression of the gluconeogenic enzyme fructose-1, 6-bisphosphotase 1 (FBP1) was reported to be repressed by Snail upon EMT in breast cancer [21], but this was not observed in our model (Fig. 2c). Surprisingly, the treatments resulted in significant and variable changes in the different isoforms of pyruvate dehydrogenase kinase (PDK 1–4) (Fig. 2i–l). Overall, Panc-1 cells displayed enhanced glucose uptake and lactate secretion upon EMT induction but showed no major changes in the levels of glycolytic enzymes.

### Oxidative metabolism was not changed upon TNFα- and TGFβ-induced EMT

**Fig. 3:**
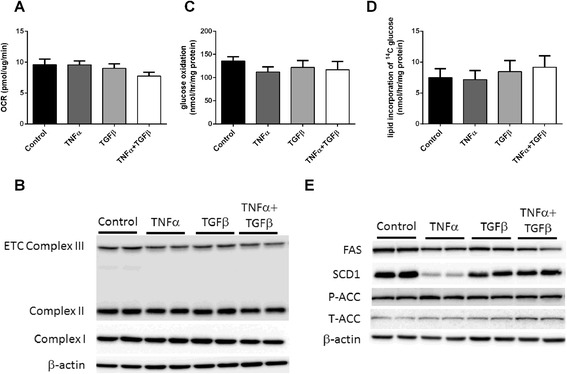
Oxidative metabolism is with TNFα- and TGFβ-induced EMT. Panc-1 cells were cultured in the presence of 40 ng/ml TNFα or 10 ng/ml TGFβ or both for 72 h to induce EMT. **a** Oxygen consumption rate (OCR) was measured using the Seahorse XF24 Bioanalyzer at basal conditions. **b** Western blot detection of mitochondrial electron transport chain complex III, II, and I. β-actin was used as a loading control. **c** Glucose oxidation was measured using the U-^14^C-glucose tracer over a 1-h period. The amount of ^14^C in CO_2_ originated from ^14^C-glucose was counted using a beta counter to give an indication of glucose oxidation rate. **d** Total lipid in the cells was extracted using the chloroform/methanol method following 1 h incubation in U-^14^C-glucose-containing media. The amount of ^14^C incorporated in the total lipid was quantified using a beta counter. **e** Western blot detection of fatty acid synthase (FAS), phospho- and total-acetyl-CoA carboxylase (P-ACC and T-ACC), and stearoyl-CoA desaturase-1 (SCD1). β-actin was used as a loading control. Results are shown as mean ± SEM with *n* = 5 (**a**) or *n* = 3 (**b**, **e**) or *n* = 4 (**c**, **d**). *p < 0.05, **p < 0.01, ***p < 0.001

Following the observation of upregulated glycolysis, we next examined the metabolic profiles of oxidative pathways. Basal oxygen consumption rate (OCR) was measured to give an overall indication of oxidative activity in these cells and was found to be unchanged upon TNFα- and TGFβ-induced EMT (Fig. 3a). The levels of protein subunits of mitochondrial electron transport chain (ETC) complexes I, II, and III also remained the same as control (Fig. 3b). To assess the oxidation of glucose, radioactive U-^14^C-glucose tracer was used, and similar to the overall oxidative metabolism, no difference was observed in glucose oxidation across the groups (Fig. 3c).

### Endogenous lipid production from glucose is unchanged upon TNFα- and TGFβ-induced EMT

Fast proliferating cells need to make membranes at a sufficient rate so the endogenous production of lipids is frequently upregulated in cancer cells [43]. When U-^14^C-glucose tracer was made available to the cells, ^14^C incorporation into total lipids did not change with any of the EMT treatments (Fig. 3d). Assessment of enzymes involved in de novo lipid synthesis, however, revealed reductions in both fatty acid synthase (FAS) and stearoyl-CoA desaturase-1 (SCD1) protein levels with all three treatments (Fig. 3e). The decrease in SCD1 following exposure to TNFα alone was the most pronounced (*p* < 0.001) (Fig. 3e). Phospho-acetyl-CoA carboxylase (ACC) (P-ACC, inactive) and total-acetyl-CoA carboxylase (T-ACC) levels did not show differences between the groups (Fig. 3e). Taken together, no difference in glucose-sourced lipogenesis was observed in TNFα- and TGFβ-induced EMT models despite some changes in lipogenic enzyme levels.

### ^13^C-Glucose tracer showed increased glucose-to-lactate conversion upon EMT induction

To confirm the changes in glycolysis and to provide a more complete picture of metabolic alterations upon EMT induction, ^13^C-glucose metabolomics experiments were carried out. Cells were treated with the respective compounds for 72 h with ^13^C-glucose replacing normal glucose in the media for the last 5 h. Samples of culture media were taken every hour for the last 5 h, and cells were collected at the end.

**Fig. 4:**
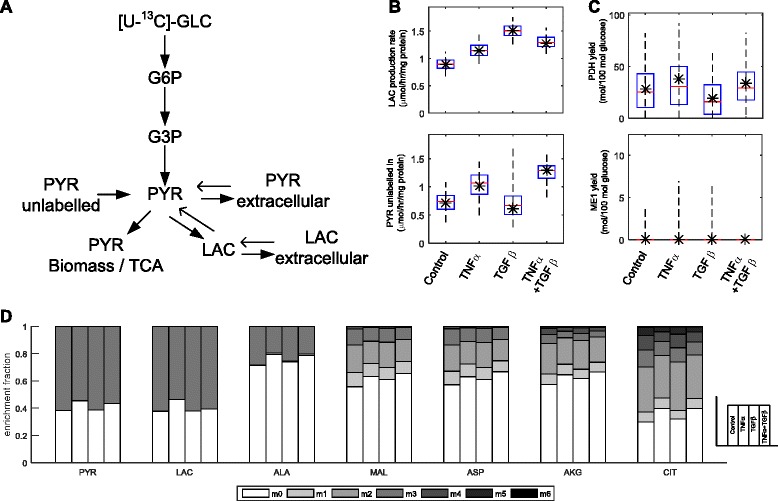
[U-^13^C]-Glucose tracer model data and flux results. Panc-1 cells were cultured in the presence of 40 ng/ml TNFα or 10 ng/ml TGFβ or both for 72 h prior to the start of the labelling experiment. **a** Metabolic model used for fitting of the extracellular enrichment data, allowing for the reversible exchange of pyruvate and lactate. *G6P* glucose 6-phosphate, *G3P* glyceraldehyde 3-phosphate. **b** Estimated rates of lactate production and uptake of non-glucose substrates from extracellular enrichment data. **c** Estimated yields of pyruvate dehydrogenase (PDH) and NADP-dependent malic enzyme (ME1) from intracellular enrichment data*. Box-and-whisker plots* show the 2.5th, 25th, 50th, 75th, and 97.5th percentile values; optimum values shown as *asterisks*. **d** Enrichment fractions of intracellular metabolites pyruvate (PYR), lactate (LAC), alanine (ALA), malate (MAL), aspartate (ASP), α-ketoglutarate (AKG), and citrate (CIT) showing the fractions of carbon atoms of these metabolites derived from glucose

Over the 5-h time course, Panc-1 cells that underwent EMT in response to treatments showed greater rates of extracellular lactate accumulation (Fig. 4b). Results were consistent with the prior lactate assay (Fig. 2b), although estimates were highest for Panc-1 cells treated with TGFβ alone. For the accumulated amount of ^13^C-enriched lactate (i.e., derived from glucose), TGFβ-treated cells showed the greatest accumulation at 6.1 mM/mg protein, whereas for control cells, it was at 3.6 mM/mg protein. For all control and treated cells at least 73 % of the lactate present extracellularly was derived from glucose (Additional file 5: Figure S3), confirming the dominant role of aerobic glycolysis in lactate production (Fig. 2).

The extracellular accumulation of enriched pyruvate was also observed, but the quantities accumulated were not different between the control and the treatments, and the concentrations accumulated were less than lactate by an order of magnitude (Additional file 5: Figure S3 ). The amount of unlabeled pyruvate, already present in the culture medium, decreased over time. This suggested the simultaneously consumption and production of extracellular pyruvate.

### No appreciable changes in TCA cycle activity

The enrichment patterns of intracellular malate, aspartate (proxy for oxaloacetate), α-ketoglutarate, and citrate were similar between the control and the EMT-induced cells (Fig. 4d). The approximately twofold increase in m0 fraction from citrate to α-ketoglutarate suggested significant glutaminolysis driving TCA cycle fluxes. Nonetheless, the extent of glutamine mixing with α-ketoglutarate remained steady across all treatments and control.

The enrichment data also suggested that cells were simultaneously decarboxylating and carboxylating pyruvate. Two-carbon acetyl-CoA, produced by pyruvate dehydrogenase, was fully catabolized, as indicated by the m2 and m4 fractions in citrate, and the propagation of these fractions to malate and aspartate. A greater m3 fraction in malate and aspartate compared to α-ketoglutarate suggested some contribution of pyruvate carboxylase towards the synthesis of oxaloacetate from pyruvate and a reversible malate dehydrogenase. Overall, all three major pathways that feed into TCA cycle, namely glutaminolysis, glycolysis, and anaplerotic flux, were actively engaged to a similar extent between the control and the EMT-induced cells.

### Contribution of alternative pathways towards lactate production

The detection of about 40 % m0 (unlabeled) fraction of intracellular pyruvate and lactate indicated significant conversion of non-glucose substrates to the pyruvate node. The production of unlabeled (natural enrichment) lactate was found to have increased in the EMT-induced cells as well, from 0.3 mM/mg protein for the control to between 1.12 and 1.75 mM/mg protein for TNFα- and TGFβ-treated cells (Additional file 5: Figure S3). The results raised the possibility that EMT may increase catabolism of alternative substrates, such as amino acids, to lactate.

It is possible that intracellular and extracellular pools of lactate and pyruvate are exchanging reversibly (i.e., well-mixed), thus contributing to the observed dilution effect. A tracer model was thus constructed to estimate the contribution of glucose and unlabeled substrates to pyruvate and lactate and, at the same time, subtracting the effects of reversible pyruvate and lactate exchange (Fig. 4a). Flux analysis was then used to calculate the contribution of non-glucose-derived substrates to the pyruvate note using the enrichment and abundance of extracellular lactate and pyruvate as constraints.

Flux results suggested that TNFα-treated cells, alone or in combination with TGFβ, showed an increased conversion of unlabeled substrates to pyruvate, with approximately the same increments propagated to the respective net lactate secretion rates (Fig. 4b). For cells treated with TGFβ alone, however, model results indicated that the high lactate production might be achieved by suppressing downstream pathways that consumed pyruvate, rather than by increasing conversion rates of glucose and unlabeled substrates. Slight downward shifts among the fluxes of pyruvate dehydrogenase (Fig. 4c) and TCA cycle enzymes, albeit not significant, were observed for the TGFβ-treated cells.

Additionally, the absence of m1 and m2 fractions of pyruvate and lactate (Fig. 4d)—intracellular and extracellular—strongly suggested the lack of NADP-dependent malic enzyme (ME1) to convert partially labelled TCA cycle intermediates into cytoplasmic pyruvate. Indeed, flux activity using the intracellular enrichment verified the lack of connectivity between TCA cycle metabolites and cytoplasmic pyruvate (Fig. 4c).

Overall, the tracer data appear to suggest that lactate production in EMT-induced cells, while predominantly attributed to glucose, also involve catabolism of alternative substrates independent of the TCA cycle.

## Discussion

Cancer cells with EMT features are observed under various experimental settings and clinical situations. In recent years, several studies have been conducted investigating the metabolic changes during EMT in breast, lung, and ovarian cancers, following an increased recognition of metabolic reprogramming as a hallmark of tumor development [20–27]. In view of the important role EMT plays in PDAC metastasis and chemoresistance, we sought to understand the metabolic adaptations underlying PDAC EMT. Here, we showed that exposure to TNFα and TGFβ, two factors commonly present in the tumor microenvironment that signal through distinct pathways, induced overt EMT in the Panc-1 cell line of human PDAC, reflected by EMT-like morphological, molecular, and functional changes. The induction of EMT by both of these factors was accompanied by augmentations of glucose uptake and lactate secretion, while no major changes to oxidative metabolism were observed. In addition, ^13^C-glucose metabolomics combined with flux analysis revealed that the increase in lactate production by these two treatments might be achieved via different mechanisms and suggested possible contributions of nutrients other than glucose.

While there is relatively limited literature in this area, the nature of the metabolic alterations appears to vary widely across different cancer types and EMT models. One of the earliest studies reported findings similar to our current results, with increased Glut1 expression and glucose uptake in the breast cancer cell line MCF-7 undergoing TGFβ-induced EMT [20]. The downregulation of gluconeogenic enzyme FBP1 was described as a predominant EMT-related change by Dong et al. [21], on the basis of comparison of luminal and the more metastatic basal subtype of breast cancer. The enhancement of glycolysis and reduction in gluconeogenesis were also observed in two breast cancer cell lines exhibiting EMT features after mammosphere cultures by Kondaveeti and colleagues, who also noted decreases in enzymes involved in the pentose phosphate and hexosamine synthesis pathways [26]. In contrast, three KRAS- or EGFR-driven non-small cell lung cancer (NSCLC) cell lines treated with TGFβ or erlotinib to induce EMT displayed reduced glycolysis to oxidation ratio and lower PDK4 expression [23]. Increased oxygen consumption rate and coordinated suppression of lipogenesis were reported in a separate study on a TGFβ-induced EMT model in NSCLC [27]. Surprisingly, increased expression of GLUT3 transporter was also associated with TGFβ treatment in the same cancer type [24]. In ovarian cancer, Aspuria et al. induced EMT via succinate dehydrogenase B knockdown and saw decreased maximal OCR, accompanied by increased glucose contribution to pentose phosphate pathway and nucleotide synthesis [22].

Physiologically, the induction of EMT is the result of contextual cues originating from surrounding cells and the circulation acting upon cancer cells that are sensitized by oncogenic mutations. Therefore, the diversity of the tumor microenvironment and genetic composition could partially underscore the heterogeneity of EMT responses. In this context, it is worth noting however that within the same cancer type, there is no current evidence that the status of common mutations appears to have a major impact on EMT-related metabolic reprogramming, as NSCLC cell lines harboring mutant or wild-type KRAS, EGFR, and TP53, as well as breast cancer cell lines with or without HER2 overexpression do not display mutation-specific changes [23, 26]. Given the complexity of genetic events in cancers, further comprehensive studies are needed to uncover any possible interactions between genomic and metabolic landscapes in relation to EMT.

The disparity of EMT metabolic reprogramming in different contexts could additionally be related to variations in the accompanying phenotypic transformations, which are potentially linked to differential metabolic modulations. For instance, stem cells exhibit heightened rates of glycolysis [44, 45] and inhibition of glycolysis was reported to suppress stemness features in glioblastoma stem-like cells [46]. Hence, the increased glycolytic flux observed in some EMT models may be related to the stemness conferred by EMT. Increases in glucose uptake would presumably promote cell survival under hypoxic and nutrient poor conditions, while the lactate secreted could facilitate matrix degradation [47] and evasion of immune surveillance [48, 49], echoing the promotion of apoptotic resistance and invasiveness by EMT. On the other hand, elevated mitochondrial biogenesis and oxidative phosphorylation have, in some cases, been associated with chemoresistance and metastatic behaviors which are also promoted by EMT [50–52].

One prominent feature of PDAC is the presence of dense stromal compartment surrounding the tumors. The dense stroma both constraints the delivery of oxygen/nutrients to the tumor cells and can also act as a physical barrier for extravasation. For PDAC cells that have undergone EMT, the increased lactate excretion may be essential for traversing the stromal layer before reaching the circulation and maintaining viability along the way. Recent reports by Fisher et al. [53] and Zheng et al. [54] questioned the role of EMT in cancer metastasis but provided in vivo evidence that EMT cells are resistant to chemotherapy. It is known that after chemotherapy or experimental induction of gemcitabine resistance, a proportion of PDAC cells exhibit EMT features and display signature stem cell surface markers [12, 55]. Treatment with the anti-glycolytic agent 3-bromopyruvate sensitized primary PDAC cancer stem cells to gemcitabine [56]. The metabolic alterations seen in PDAC cells in the present study are consistent with an increase in stem-like properties with EMT, which could contribute to chemoresistance.

Pinpointing the molecular changes underlying the observed metabolic reprogramming with EMT is important for developing targeted therapies. It could be reasoned that increased lactate output was driven by increased expression of GLUT transporters (1 and 3) and the consequent increase in glycolytic influx, as no major changes in glycolytic and lactate-producing enzymes were observed. However, ^13^C enrichment data suggested that lactate was simultaneously produced from both glucose and non-glucose sources, albeit the former contributing to the majority. While glutamine could act as a significant non-glucose substrate for cancer cells, data from radioactive and stable tracer experiments revealed that neither the TCA cycle activity was enhanced nor its intermediates contributed to lactate production. Further research will therefore be needed to identify the full spectrum of lactate precursors in the EMT models when strategies to “starve” the malignant cells are pursued. In addition, enrichment data from TGFβ-treated cells raised the prospect that a greater pyruvate-to-lactate conversion during EMT could also be achieved by downregulating pyruvate consumption pathways, e.g., PDH. The observed changes of PDK isoforms, which control the partitioning of pyruvate to the TCA cycle, were disparate, possibly providing a mechanism of fine tuning glucose metabolism.

## Conclusions

Our study has revealed increases in glucose uptake and lactate secretion as prominent metabolic adaptations of two models of EMT in a human PDAC cell line. Understanding of the metabolic reprogramming associated with EMT in PDAC provides evidence for developing metabolic strategies to selectively target EMT cells and to avoid the accidental promotion of EMT in cancer cell populations while applying metabolic therapies against proliferating cells. The differences in the underlying molecular changes seen in the two EMT models in the current study and other EMT models in various cancer types further highlight the heterogeneity of cancers and therefore a need for context-specific investigations of tumor metabolism.

## Additional files

Additional file 1: Table S1. Sources and ID numbers of qPCR primers. (DOCX 14 kb)
Additional file 2: Figure S1. Wound healing assay images for Fig. 1f. (DOCX 447 kb)
Additional file 3: Figure S2. Steady state yield estimates generated from ^13^C enrichment data of intracellular metabolites using OpenFLUX. (DOCX 358 kb)
Additional file 4: OpenFLUX model for extracellular metabolites. (XLSX 12 kb)
Additional file 5: Figure S3. Extracellular lactate and pyruvate labelled by [U-^13^C]-glucose. (DOCX 3306 kb)

## Abbreviations

2-DG: 2-Deoxyglucose
ACC: Acetyl-CoA carboxylase
EMT: Epithelial-mesenchymal transition
ETC: Electron transport chain
FAS: Fatty acid synthase
FBP1: Fructose-1, 6-bisphophotase 1
HK2: Hexokinase II
LDH: Lactate dehydrogenase
MCT: Monocarboxylic acid transporter
OCR: Oxygen consumption rate
PDAC: Pancreatic ductal adenocarcinoma
PDK: Pyruvate dehydrogenase kinase
PKM2: Pyruvate kinase isoform 2
SCD1: Stearoyl-CoA desaturase-1
TGFβ: Transforming growth factor-β
TNFα: Tumor necrosis factor-α
