# Supplementary material for: Epithelial-mesenchymal transition induction is associated with augmented glucose uptake and lactate production in pancreatic ductal adenocarcinoma
Source: Cancer Metab. 2016 Oct 17;4:19. doi: 10.1186/s40170-016-0160-x (PMC5066287; doi:10.1186/s40170-016-0160-x)
Supplement: Additional file 1: Table S1. — Sources and ID numbers of qPCR primers. (DOCX 14 kb) [file 40170_2016_160_MOESM1_ESM.docx]

**Additional file 1. Table S1.** Sources and ID numbers of qPCR primers

| Transcript | source | ID |
| --- | --- | --- |
| SNAI1 | primer bank | 301336132c1 |
| SNAI2 | primer bank | 324072669c1 |
| SLC2A1 | Sigma Kicqstart SYBR qPCR | H1_SLC2A1 |
| SLC2A3 | Sigma Kicqstart SYBR qPCR | H1_SLC2A3 |
| LDH-B | Sigma Kicqstart SYBR qPCR | H_LDHB_1 |
| MCT1 | Sigma Kicqstart SYBR qPCR | H_SLC16A1_1 |
| MCT4 | Sigma Kicqstart SYBR qPCR | H_SLC16A4_1 |
| PDK1 | Sigma Kicqstart SYBR qPCR | H_PDK1_1 |
| PDK2 | Sigma Kicqstart SYBR qPCR | H_PDK2_1 |
| PDK3 | Sigma Kicqstart SYBR qPCR | H_PDK3_1 |
| PDK4 | primer bank | 94421466c1 |
| b-actin | primer bank | 4501885a1 |
