# Supplementary material for: Epithelial-mesenchymal transition induction is associated with augmented glucose uptake and lactate production in pancreatic ductal adenocarcinoma
Source: Cancer Metab. 2016 Oct 17;4:19. doi: 10.1186/s40170-016-0160-x (PMC5066287; doi:10.1186/s40170-016-0160-x)
Supplement: Additional file 2: Figure S1. — Wound healing assay images for Fig. 1f. (DOCX 447 kb) [file 40170_2016_160_MOESM2_ESM.docx]

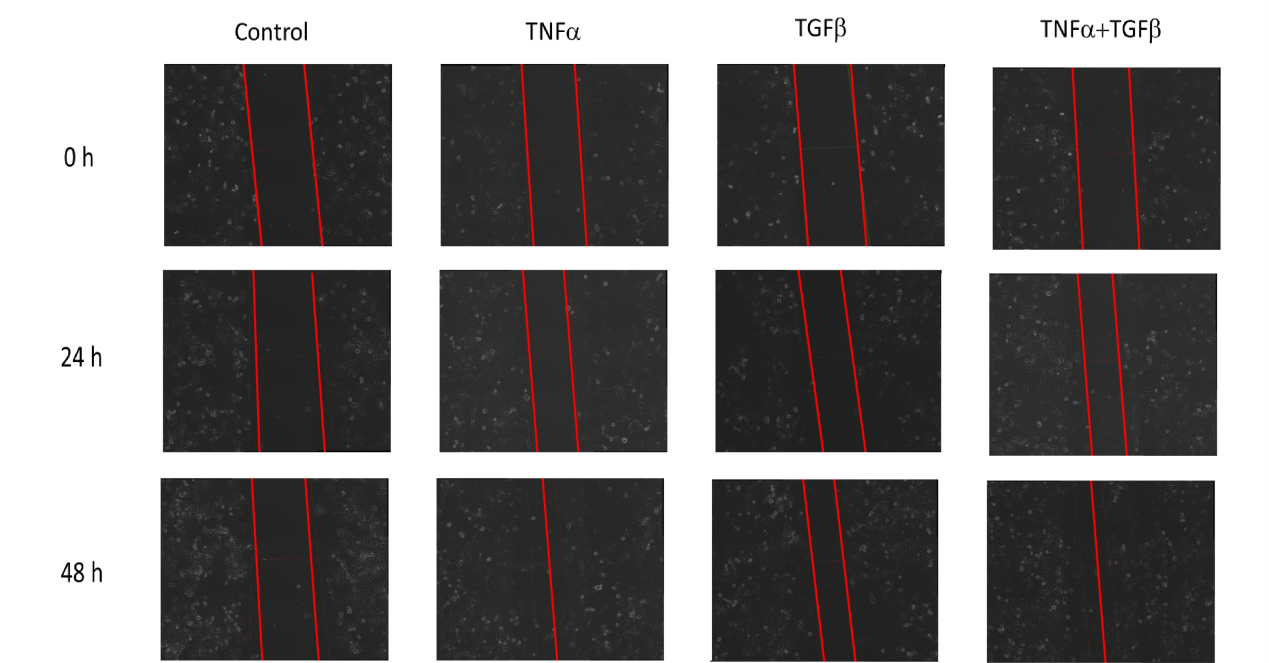


**Additional file 2. Figure S1.** Cells were seeded onto 6-well plates and allowed to grow to 50% confluence in the presence of added factors for 2 days. The monolayer was wounded with a plastic tip and monitored under bright field microscope for 48 hours with continued treatments with images taken at 0, 24 and 48 hrs.
