# Supplementary material for: Epithelial-mesenchymal transition induction is associated with augmented glucose uptake and lactate production in pancreatic ductal adenocarcinoma
Source: Cancer Metab. 2016 Oct 17;4:19. doi: 10.1186/s40170-016-0160-x (PMC5066287; doi:10.1186/s40170-016-0160-x)
Supplement: Additional file 3: Figure S2. — Steady state yield estimates generated from 13C enrichment data of intracellular metabolites using OpenFLUX. (DOCX 358 kb) [file 40170_2016_160_MOESM3_ESM.docx]

**Additional file 3. Figure S2.** Steady-state yield estimates (mol per 100 mol glucose) generated from 13C enrichment data of intracellular metabolites using OpenFLUX. 1 – Control; 2 – TNFα; 3 – TGFβ; 4 – TNFα + TGFβ. (A) Least-square estimates of fluxes showing optimum and Monte-Carlo (1000 iteration) results. Stars represent optimum values, box-whisker plots show 2.5^th^, 25^th^, 50^th^, 75^th^ and 97.5^th^ percentile values. (B) Metabolic model used for flux estimates. Experimental (left) and simulated (right) enrichment data shown as stacked bar graphs. Reaction numbers (e.g. R1) are detailed in Additional file 5.
