# Supplementary material for: Epithelial-mesenchymal transition induction is associated with augmented glucose uptake and lactate production in pancreatic ductal adenocarcinoma
Source: Cancer Metab. 2016 Oct 17;4:19. doi: 10.1186/s40170-016-0160-x (PMC5066287; doi:10.1186/s40170-016-0160-x)
Supplement: Additional file 5: Figure S3. — Extracellular lactate and pyruvate labelled by [U-13C]-glucose. (DOCX 3306 kb) [file 40170_2016_160_MOESM5_ESM.docx]

**Additional file 5. Figure S3. Extracellular lactate and pyruvate labelled by [U-13C]-glucose**

Panc-1 cells were cultured in the presence of 40 ng/ml TNFα or 10 ng/ml TGFβ or both for 72 hours prior to the start of the labelling experiment. Concentration of extracellular lactate and pyruvate showing the different enrichment fractions m0 (unlabelled), m1, m2 and m3 (fully labelled). Shaded bars show accumulation of lactate and pyruvate derived from glucose. m1 and m2 of pyruvate omitted due to isobaric interference. Overlaying trend lines show the performance of a Monte-Carlo least-square flux analysis approach to fit the dataset (1000 iterations).
